# Loop-Mediated Isothermal Amplification Assay for the Rapid Detection of *Staphylococcus aureus*

**DOI:** 10.1155/2013/895816

**Published:** 2013-01-15

**Authors:** King Ting Lim, Cindy Shuan Ju Teh, Kwai Lin Thong

**Affiliations:** ^1^Institute of Biological Science, Faculty of Science, University of Malaya, 50603 Kuala Lumpur, Malaysia; ^2^Laboratory of Biomedical Science and Molecular Microbiology, Institute of Graduate Studies, University of Malaya, 50603 Kuala Lumpur, Malaysia

## Abstract

*Staphylococcus aureus*, including methicillin-resistant *S. aureus* (MRSA), is an important human pathogen that produces a variety of toxins and causes a wide range of infections, including soft-tissue infections, bacteremia, and staphylococcal food poisoning. A loop-mediated isothermal amplification (LAMP) assay targeting the *arcC* gene of *S. aureus* was developed and evaluated with 119 *S. aureus* and 25 non-*S. aureus* strains. The usefulness of the assay was compared with the PCR method that targets *spa* and *arcC* genes. The optimal temperature for the LAMP assay was 58.5°C with a detection limit of 2.5 ng/**μ**L and 10^2^ CFU/mL when compared to 12.5 ng/**μ**L and 10^3^ CFU/mL for PCR (*spa* and *arcC*). Both LAMP and PCR assays were 100% specific, 100% sensitive, 100% positive predictive value (PPV), and 100% negative predictive value (NPV). When tested on 30 spiked blood specimens (21 MRSA, eight non-*S. aureus* and one negative control), the performance of LAMP and PCR was comparable: 100% specific, 100% sensitive, 100% PPV, and 100% NPV. In conclusion, the LAMP assay was equally specific with a shorter detection time when compared to PCR in the identification of *S. aureus*. The LAMP assay is a promising alternative method for the rapid identification of *S. aureus* and could be used in resource-limited laboratories and fields.

## 1. Introduction

*Staphylococcus aureus,* including methicillin-resistant *S. aureus* (MRSA), is an important bacterial pathogen associated with community and healthcare *S. aureus* infections in Malaysia and worldwide. They are known to produce a variety of virulence factors, including the staphylococcal enterotoxins (SEs) and the toxic shock syndrome toxin (TSST) which are responsible for staphylococcal food poisoning [1, 2].

Polymerase chain reaction (PCR) and real-time PCR have been used for rapid identification of *S. aureus*, particularly for MRSA [3, 4]. Both methods require the use of special equipment, that is, PCR thermocycler or real-time PCR, respectively.

 Loop-mediated isothermal amplification (LAMP) which is based on autocycling strand displacement DNA synthesis using the *Bst* DNA polymerase enzyme was developed by Notomi et al. [5]. This *Bst* DNA polymerase is derived from *Bacillus stearothermophilus*, which possesses a 5′→3′ exonuclease activity that needs high concentration of magnesium for optimum activity [6]. This enzyme can be inactivated by incubation at 80°C for 15 min. In the LAMP method, four (B3, F3, FIP, and BIP) to six (B3, F3, FIP, BIP, Loop-F, and Loop-R) primers are used for amplification, and the reactions are carried out at isothermal condition [7, 8]. Loop primers that bind to loop structures are used to shorten the reaction time of the LAMP assay [9]. The LAMP assay has been developed for rapid identification of a wide variety of bacteria, including *S. aureus, Vibrio parahaemolyticus*, *Campylobacter jejuni, Campylobacter coli, Leptospira* species, *Salmonella *Typhi, and *Escherichia coli* [10–15]. 

The objective of this study was to develop and determine the usefulness of the LAMP assay in comparison with PCR for rapid identification of *S. aureus*.

## 2. Materials and Methods

### 2.1. Bacterial Isolates

 A well-characterized strain of* S. aureus* (MRSA0807-7) with genotype MLST type ST239, SCC*mec* type III, and* spa* type t421 or also known as ST239-III-t421 [16] was used for the optimization of LAMP assay. The sensitivity and specificity of the assay were evaluated with 124 clinical bacterial strains, which included 79 methicillin-resistant *S. aureus *(MRSA), 20 methicillin-sensitive *S. aureus *(MSSA), 5 *Staphylococcus epidermidis*, 5 *Salmonella* Typhimurium, 5 *Shigella sonnei*, 5 *Listeria monocytogenes*, and 5 *Escherichia coli*. 

All MRSA and MSSA strains were previously isolated from inpatients admitted to a local tertiary hospital. The MSSA strains were previously confirmed by BBL Staphyloslide-Latex Test (BD, USA) [17] while MRSA strains were confirmed by standard biochemical tests [16] with minor modifications. In brief, all MRSA strains were streaked on blood and mannitol salt agars and incubated overnight at 35°C. Strains that showed *β*-hemolysis in blood agar and appeared as yellow colonies in mannitol salt agar were subjected to coagulase test and cefoxitin disk diffusion test. The strain was confirmed as MRSA strain if it gave a positive result in the coagulase test and has zone diameter of less 22 mm in cefoxitin disk diffusion test [16]. 

All MSSA strains were isolated from nasal swabs whereas MRSA strains were isolated from nasal swabs (*n* = 40), sputum (*n* = 20), wound swabs (*n* = 9), urine (*n* = 5) and body fluids (*n* = 5). 

### 2.2. Preparation of DNA Template

Crude DNA from Gram-negative bacteria such as *E. coli, Salmonella *Typhimurium, and *Shigella sonnei* was obtained by direct boiled cell lysate. Briefly, a loopful of bacterial culture was mixed with 100 *μ*L of sterile-deionized water, and the suspension was boiled in 100°C for 5 min. After boiling, the cell lysate was snapped cool for 5 min in ice and centrifuged at 10,000 ×g for 2 min. The supernatant was transferred to a clean microfuge tube and used as DNA template for LAMP and PCR analyses. 

Crude DNA from Gram-positive *S. aureus* and* S. epidermidis *was obtained by using lysostaphin-lysis method while crude DNA for *L. monocytogenes* was obtained by using a lysozyme-lysis method. Procedures for lysostaphin and lysozyme-lysis methods were very similar to the boiling method except for the addition of lysostaphin (2 *μ*g/mL) or lysozyme (20 *μ*g/mL) in the bacterial culture and incubation at 37°C for 10 min. The DNA was quantified by using Eppendorf BioPhotometer (Eppendorf Ltd, Germany). 

Meanwhile, crude DNA from spiked blood samples was obtained by centrifugation and washing the residue twice with deionized water followed by direct cell lysis. Briefly, 100 *μ*L of spiked blood sample was mixed with 900 *μ*L of sterile deionized water and the suspension was centrifuged at 10,000 ×g for 3 min. The supernatant was discarded and the pellet was resuspended in 1 mL of sterile deionized water before they were centrifuged at 10,000 ×g for 3 min. The supernatant was removed and the pellet was mixed with 300 *μ*L sterile deionized water. Lysostaphin (2 *μ*g/mL) was added to the cell suspension and was incubated at 37°C for 5 min, followed by heating at 100°C for 10 min. After boiling, the cell lysate was snapped cool for 5 min in ice. The cell lysate was then centrifuged and the supernatant was transferred to a clean microfuge tube and used as a DNA template. 

### 2.3. Primer Design of the *arcC* Gene for LAMP Assay

Carbamate kinase gene (*arcC*), one of the housekeeping genes used for multilocus sequence typing (MLST) of *S. aureus, *was selected for this study [18]. Two pairs of primers, including F3 (5′-GTCTTTAAAGAAGATGCAGGAC-3′), B3 (5′-GCGTTGCTAATTTCTCACT-3′), forward inner primer (FIP) (ACCGTCTGCTAAAGTTCGAATTAAC-TAGTTGCGTCACCACTAC), and backward inner primer (BIP) (TGGTGGCGGTATTCCAGTTA-ATAACCGCTTCAACACCTTC), were designed by using online PrimerExplorer V4 program (PrimerExplorer, Eiken Chemical Co. Ltd.). 

### 2.4. Optimization of the LAMP Assay Using Different Temperatures

Optimization of the LAMP assay was performed on a reference strain MRSA0807-7 by using Loopamp DNA amplification kit (Eiken Chemical Co. Ltd., Tokyo, Japan). The kit is based on the use of four kinds of primers (two inner and two outer) that recognize six distinct regions of a target gene in the presence of *Bst *DNA polymerase with strand-displacement activity along with substrates, mixture of samples with incubation at a constant temperature (http://www.loopamp.eiken.co.jp/e/lamp/index.html).

Briefly, 25 *μ*L of the reaction mixture containing 4 *μ*L of each FIB and BIP primer (equivalent to 40 pmol of each FIB and BIP primer), 0.5 *μ*L of each F3 and B3 primer (equivalent to 5 pmol of each F3 and B3 primer), 12.5 *μ*L of 2X reaction mixture (provided in the kit), 1 *μ*L of *Bst* DNA polymerase (provided in the kit), 1.5 *μ*L of sterile deionize water, and 1 *μ*L of DNA template (5 ng) was used. 

The reaction mixtures were incubated at different temperatures ranging from 56°C to 63°C for 80 min, followed by enzyme inactivation at 80°C for 2 min in the Loopamp real-time turbidimeter (LA-320, Teramecs, Co., Ltd., Kyoto, Japan). This Loopamp real-time turbidimeter is specifically designed for real-time monitoring of the LAMP reaction. The reaction is considered positive when the turbidity reached 0.1 within 60 min at 650 nm. The time needed for the turbidity of each tested sample to exceed OD_650 nm_ at 0.1 is referred to as the threshold time (Tt) [19]. 

### 2.5. Evaluation of the LAMP Assay on Bacterial Cultures

The optimized LAMP assay was performed on all 79 MRSA, 20 MSSA, 5 *S. epidermidis*, 5 *Salmonella* Typhimurium, 5 *Shigella sonnei*, 5 *E. coli*, and 5 *L. monocytogenes* by using Loopamp DNA amplification kit (Eiken Chemical Co. Ltd., Tokyo, Japan). Following the optimization temperature (refer to result), the amplification of LAMP assay was performed at 58.5°C for 80 min and followed by 80°C for 2 min. 

In addition, a positive result could also be determined by direct visualization of turbidity of the mixtures or by the formation of a white precipitate at the bottom of the microfuge tube after centrifugation at 10,000 ×g at 3 min. 

### 2.6. PCR Detection of *spa* and *arcC* Genes

In parallel, PCR targeting *spa* and *arcC* genes was performed on 79 MRSA, 20 MSSA, 5 *S. epidermidis*, 5 *E. coli*, 5 *Salmonella* Typhimurium, 5 *Shigella sonnei*, and 5 *L. monocytogenes* as previously described by Harmsen et al. [20] and Enright et al. [18], respectively. Briefly, two Monoplex PCR were carried out in a 25 *μ*L volume containing 1.4 mM MgCl_2_, 35 mM each deoxynucleoside triphosphate, 0.3 *μ*M of each 1095F and 1517R primers (for *spa* gene) or 0.3 *μ*M of each arcC-1 and arcC-2 primers (for *arcC* gene), 1X Green buffer, 0.5 U *Taq* DNA polymerase (Promega, Madison WI, USA), and 5 *μ*L of DNA template. Following PCR, 5 *μ*L aliquots of each sample were subjected to electrophoresis on 1.5% agarose gels. Representative amplicons of *spa* and *arcC* genes were purified by using Qiagen DNA purification kit (Qiagen GmBH, Germany) and sequenced to validate their identities. 

### 2.7. Determination of the Detection Limit (Sensitivity) in LAMP and PCR Assays Using Pure Culture

To determine the detection limit (sensitivity) of LAMP and PCR assays, DNA template of MRSA0807-7 was serially diluted 10-fold with sterile water to 10^−1^ to 10^−8^ concentrations. One *μ*L of genomic DNA from each dilution was used as a DNA template for the LAMP and another 5 *μ*L of genomic DNA was used as a DNA template for the PCR assay.

To determine the detection limit in terms of CFU, a 6 hr culture of MRSA0807-7 was serially diluted 10-fold with 0.85% normal saline. An aliquot of 100 *μ*L of each dilution was plated on a Tryptone Soy Agar (TSA) plate to enumerate the bacterial count and another 100 *μ*L was used for DNA preparation for LAMP and PCR tests.

### 2.8. Preparation of Spiked Blood Samples and LAMP Assay

Blood samples used in this study were obtained from a healthy donor. The blood samples were stored at 4°C in K2-EDTA anticoagulated blood tube. Different microorganisms, including 21 MRSA, 4 Salmonella Typhimurium, 2 Klebsiella pneumoniae, 2 *E. coli*, and 1 sterile distilled water without bacterial DNA were used for sensitivity and specificity evaluation. 

An aliquot of 100 *μ*L of a 6 hr culture growth (OD_600 nm_ at 1) was serially diluted 10-fold dilutions in 0.85% of normal saline. An aliquot of 100 *μ*L of each dilution was plated on a Tryptone Soy Agar (TSA) plate to enumerate the bacterial count. In parallel, an aliquot of 100 *μ*L of each dilution was spiked into a 900 *μ*L of blood samples and incubated at 37°C overnight. An aliquot of 100 *μ*L of spiked blood samples was then plated on TSA plate for the determination of CFU count and another 100 *μ*L was used for DNA preparation as described earlier.

## 3. Results

### 3.1. Optimization of the Conditions for the LAMP Assay

Crude DNA from the reference MRSA0807-7 strain was used as the target template in order to determine the optimal condition for the LAMP assay. Amplification with primers F3, B3, FIP, and BIP by LAMP assay yielded a positive result at 58 min under isothermal condition of 60°C when measured by using real-time turbidimeter (LA-320, Teramecs, Co., Ltd., Kyoto, Japan). No difference was observed when the LAMP assays were performed under isothermal condition between 60°C and 62°C. No amplification was observed at 63°C.

 The product amplified at 58.5°C yielded positive result at 52 min when compared to other temperatures. Although a positive reaction was detected using different reaction temperatures between 60°C and 62°C, we found out that 58.5°C was the optimum temperature for the LAMP assay since positive reaction was detected at 52 min when compared to 58 min using reaction temperatures of 60°C to 62°C. Hence, a reaction temperature of 58.5°C was selected as the optimum temperature for LAMP assay.

### 3.2. PCR Detection of *spa* and *arcC* Genes

All 79 MRSA and 20 MSSA strains were tested positive for *spa* and* arcC* genes. Further sequencing of *spa *and *arcC* amplicons revealed that all 79 MRSA and 20 MSSA strains were confirmed as *S. aureus*. No amplification was observed for 25 other non-*S. aureus* microorganisms. 

### 3.3. Detection Limit (Sensitivity) and Specificity of LAMP and PCR Assays

The sensitivity of LAMP and PCR assays was done by using both template DNA and minimal CFU of bacteria. The detection limit for the LAMP assay was 2.5 ng/*μ*L and 10^2^ CFU/mL while the detection limit for the PCR assay was at 12.5 ng/*μ*L (since 5 *μ*L of DNA template was used in the PCR assay when compared to 1 *μ*L of DNA template used in the LAMP assay) and 10^3^ CFU/mL. 

As for the specificity test, LAMP assays were performed on all 124 isolates including MRSA, MSSA, *S. epidermidis*, *L. monocytogenes*, *Salmonella* Typhimurium, and *Shigella sonnei*. All 99 *S. aureus* strains gave positive results in LAMP assay with Tt values of 52 min 48 sec to 55 min. No Tt value was observed for all non-*S. aureus* strains indicating negative results. 

After centrifugation, all the reaction tubes that contained MRSA and MSSA showed a white precipitate at the bottom of the tube. No white precipitate was observed in tubes containing *E. coli, S. epidermidis, Shigella sonnei*, *L. monocytogenes*, and *Salmonella *Typhimurium.

### 3.4. Sensitivity and Specificity of the LAMP Assay and PCR on Spiked Blood Samples

Thirty known bacterial cultures were blind-coded and spiked in blood samples. These samples were tested with the optimized LAMP and PCR assays. 

Different concentrations of bacterial cell cultures ranging from of 1.5 × 10^2^ CFU/mL to 1.5 × 10^8^ CFU/mL were spiked into each blood sample. The minimal detectable concentration for LAMP was 1.76 × 10^2^ CFU/mL and for PCR was 1.76 × 10^3^ CFU/mL. 

Twenty-one *S. aureus *strains yielded positive results with Tt values between 53 and 55 min while non-*S. aureus* strains yielded negative results.

The data was compared with PCR results and the identity of the strains. Both LAMP and PCR were 100% specific, 100% sensitive, 100% positive predictive value (PPV), and 100% negative predictive value (NPV). 

## 4. Discussion

*S. aureus, *including MRSA, is a persistent human pathogen responsible for a variety of infections ranging from soft-tissue infections to bacteremia [21]. Thus, an accurate and rapid detection of *S. aureus* is much needed to reduce the risk factor caused by this organism. The identification of *S. aureus* by a conventional bacterial culture test often requires 1 to 2 days with the plating on blood agar and a series of biochemical tests, including the coagulase test. Although PCR assay shortens the identification time to 4 to 5 hours, this technique requires special equipment such as PCR thermocycler, electrophoresis set, and gel documentation system. 

In this study, LAMP assay was used for the rapid identification of *S. aureus* from pure cultures and spiked blood specimens. Two pairs of primers (inner and outer) were used in the LAMP assay, and the whole reactions occur in a single tube containing of *Bst* DNA polymerase, DNA templates, and reaction buffers under a constant temperature. Therefore, denaturation of DNA template could be omitted in the LAMP assay. 

The amplicons from the LAMP assay could be quantitatively profiled and measured by using the Loopamp turbidity meter which revealed the amplification kinetics of the tested strains. We also found out that the LAMP assay performance could be carried out at wider reaction temperatures ranging from 58°C to 62°C using 60 min as the cut-off time (standard isothermal amplification protocol).

In this study, Loopamp real-time turbidimeter was used in the LAMP assay whereas PCR thermocycler, gel electrophoresis, and gel documentation systems are needed for PCR analysis. Even though both approaches use special equipment, the LAMP assay holds an advantage as the positive result of the LAMP assay could be viewed through the Loopamp real-time turbidimeter screen. Furthermore, Loopamp real-time turbidimeter was set to measure the concentration of the tube at six-second intervals, and therefore, we are able to confirm the amplification threshold time on the screen even while the reaction is still ongoing. Unlike LAMP, the PCR assay requires conventional PCR amplification using a PCR thermocycler followed by agarose gel electrophoresis and staining with either ethidium bromide or gel-red before we are able to view the result through the gel documentation system. Therefore the endpoint detection of the LAMP assay is simpler and more direct. 

Although both LAMP and PCR assays showed 100% specificity, the LAMP assay produced higher sensitivity when compared to the PCR assay as the detection limit of the LAMP assay was 2.5 ng/*μ*L which was approximately five times more sensitive than PCR assay. 

The whole process of the LAMP assay from the preparation of DNA template to endpoint detection only required 80 min even whereas the conventional PCR required 4 hours starting from DNA template to the visualization of the amplicons using agarose gel electrophoresis. Furthermore, the present LAMP method also allowed rapid identification of* S. aureus *in positive blood samples within 80 min which is shorter than 2 hours as reported earlier by Misawa et al. [22]. The detection limit of LAMP assay using the spiked blood sample was comparable with pure cultures. This is not surprising as Kaneko et al. [23] have reported that LAMP assay is more tolerant towards inhibitory substances in the clinical samples. However, Wang et al. [24] reported that the usefulness of the LAMP assay could be limited by the presence of inhibitors in raw milk.

In addition, positive results could be observed after 52 min even without the use of loop primers indicating that it was not mandatory to include loop primers in the assay. This reaction time was shorter than the 60 min reported by Wang et al. [15] since the whole reaction of the LAMP assay was carried out under isothermal condition; time loss due to thermal changes could be prevented. 

Another advantage of this LAMP assay was that it only requires a heating block, which is easily available in most laboratories. Furthermore, the result or product from the LAMP assay could be determined by unaided eye without requiring special equipment. White precipitate could also be easily seen at the bottom of the tube after centrifugation at 10,000 ×g at 3 min indicating the positive result. These white precipitates contained white magnesium pyrophosphate, which is generated during the process of strand displacement of auto-cycling reaction [14].

 In conclusion, the LAMP assay was equally specific when compared to PCR in the identification of *S. aureus. *By using optimum conditions, it is not necessary to include loop primers in the lamp assay. LAMP assay is a rapid, flexible, and simple tool for the identification of *S. aureus* isolates. 
